# Reduction in far-field potentials and an accurate depiction of conduction gaps using close unipolar electrograms with an ultra-high-resolution mapping catheter

**DOI:** 10.1016/j.hrcr.2024.12.009

**Published:** 2024-12-19

**Authors:** Takehiro Nomura, Yosuke Mizuno, Daiki Kumazawa, Kosuke Onodera, Shigeru Toyoda, Kennosuke Yamashita

**Affiliations:** 1 Heart Rhythm Center, Department of Cardiovascular Medicine, Sendai Kosei Hospital, Miyagi, Japan; 2 Department of Cardiovascular Medicine, School of Medicine, Dokkyo Medical University, Shimotsuga-gun, Tochigi, Japan

**Keywords:** Catheter ablation, 3-Dimentional mapping system, Close unipolar electrogram, Far-field potential, Ultra-high-resolution mapping catheter, Conduction gap, Blockline, Local activation time

**Key Teaching Points**

• The close mapping reference electrode embedded in the catheter shaft (TRUEref technology) significantly reduced large, sharp far-field potentials.
• In areas with low voltage and complex atrial electrograms, it accurately localized block lines and gaps along the ablation line without requiring manual annotation. Accurate depiction of conduction gaps using TRUEref can support targeted ablation strategies, as demonstrated by the successful re-isolation of the right pulmonary vein in this case.
• These findings underscore the importance of advanced mapping technologies, such as TRUEref, in enhancing the precision and effectiveness of atrial tachycardia treatment.

## Introduction

There are primarily 2 important aspects in identifying tachycardia circuits: (1) Precisely exclude any other tachycardias that coexist during the mapping of the targeted tachycardia and mechanical stimulation from catheters^1^; (2) Accurately annotate complex potentials without incorrect annotations. The CARTO 3D mapping system (Biosense Webster, Diamond Bar, CA) uses a wavefront annotation algorithm that automatically annotates the peak negative slopes (–dV/dT) of the unipolar electrograms, making the accuracy of the unipolar potentials critically important.^2^ Unlike the bipolar electrograms, The Wilson Central Terminal (WCT) is generally used as a reference for unipolar electrocardiograms, but it sometimes results in incorrect annotations because of far-field and nonphysiologic signal noise.

The OCTARAY and Optrell mapping catheters (Biosense Webster, Diamond Bar, CA) have a close indifferent electrode embedded in the catheter shaft (TRUEref Technology).^3^ The close unipolar electrocardiogram is expected to reduce far-field potentials and incorrect annotations and more clearly visualize critical isthmus sites of complex atrial tachycardias.^3^^,^^4^ However, to the best of our knowledge, there have been few clinical reports on the usefulness of the TRUEref technology for assessing the tachycardia circuit.

## Case presentation

**Figure 1:**
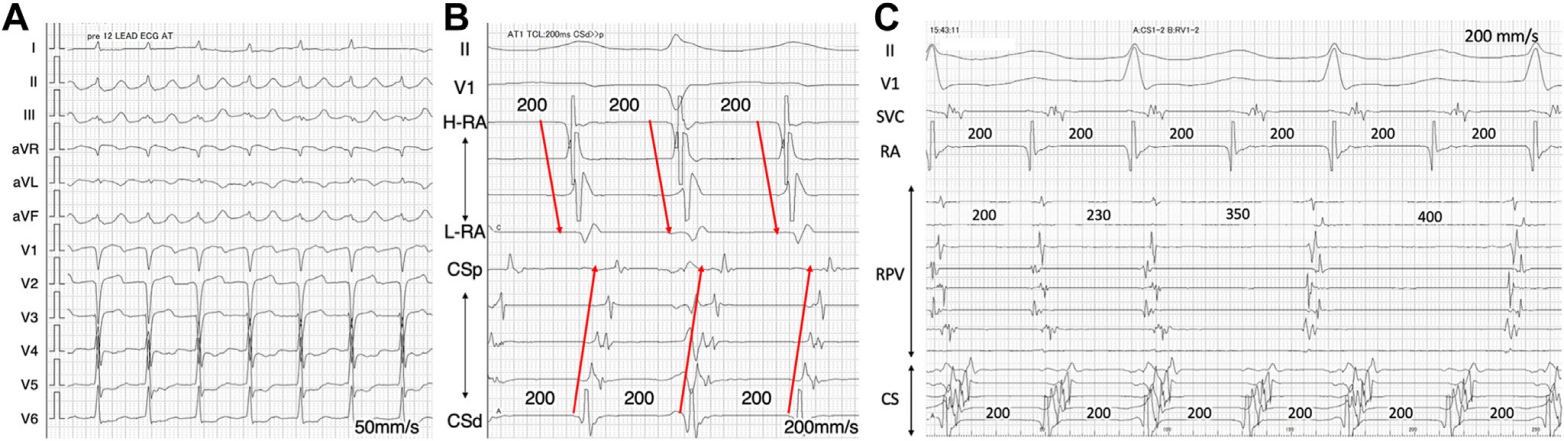
The electrocardiogram shows an atrial tachycardia with a tachycardia cycle length of 200 msec (**A, B**). The coronary sinus activation pattern is distal to proximal (**B**). Two-to-one conduction to the right pulmonary vein (RPV) is observed, and the RPV was not included in the reentrant circuit because disassociated activity was identified in the RPV (**C**).

A 71-year-old man who underwent radiofrequency catheter ablation of persistent atrial fibrillation when he was 67 years old, during which pulmonary vein isolation and cavotricuspid isthmus ablation were performed, underwent a second procedure for a recurrent atrial tachycardia (AT). The session was performed using an open-irrigated contact-force sensing catheter (Qdot Micro catheter, Biosense Webster, Diamond Bar, CA) with an electroanatomic mapping system (CARTO3, Biosense Webster, Diamond Bar, CA) under general anesthesia. A 6-Fr 20-pole electrode catheter (BeeAT; Japan Lifeline, Tokyo, Japan) was inserted through the right jugular vein into the coronary sinus (CS) as a reference to obtain dual-chamber unipolar electrograms (EGMs) from the CS and right atrium. The intracardiac EGMs were filtered between 16 and 300 Hz for the unipolar EGMs using a CARTO 3D mapping system. The atrial tachycardia cycle length was 200 milliseconds, and the CS activation pattern had a distal to proximal sequence (Figure 1B). Ultra-high-resolution mapping using the OCTARAY with 2.0-cm splines and a 3-3-3-3-3-mm interelectrode spacing suggested that the tachycardia circuit was a left atrial (LA) roof-dependent AT. A conduction gap in the right pulmonary vein (RPV) was also observed, but the RPV was not included in the reentrant circuit because disassociated activity was identified in the RPV (Figure 1C).

**Figure 2:**
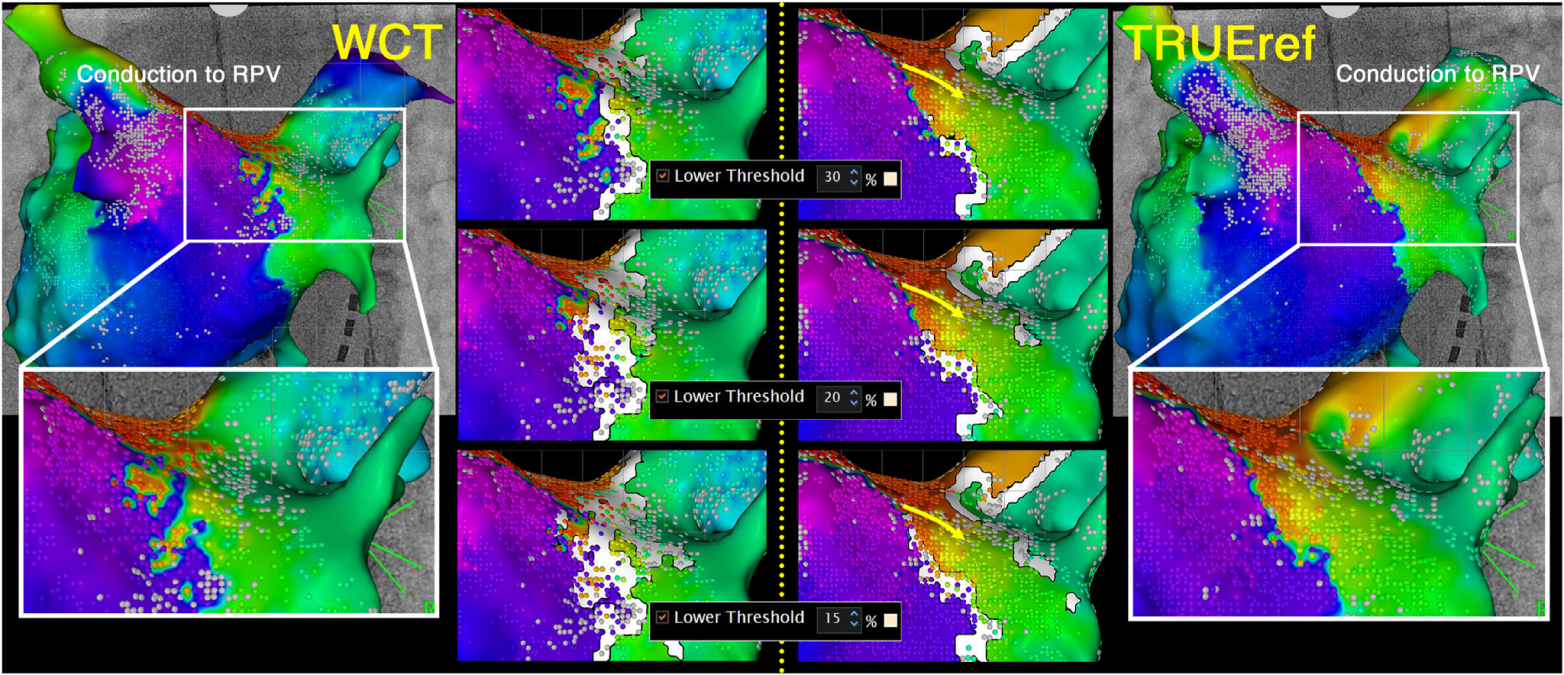
Activation maps of the left atrium using the Wilson Central Terminal (WCT) and the close unipolar reference electrode (TRUEref) were obtained. Conduction block around the right pulmonary vein (RPV) was more clearly delineated with the TRUEref than WCT. The activation map using WCT and the extended early meets late (EEML) technology exhibited a gap depicted as a conduction block. However, the map with the TRUEref and EEML showed the gap more clearly when the lower threshold was 15% than 20% or 30%.

**Figure 3:**
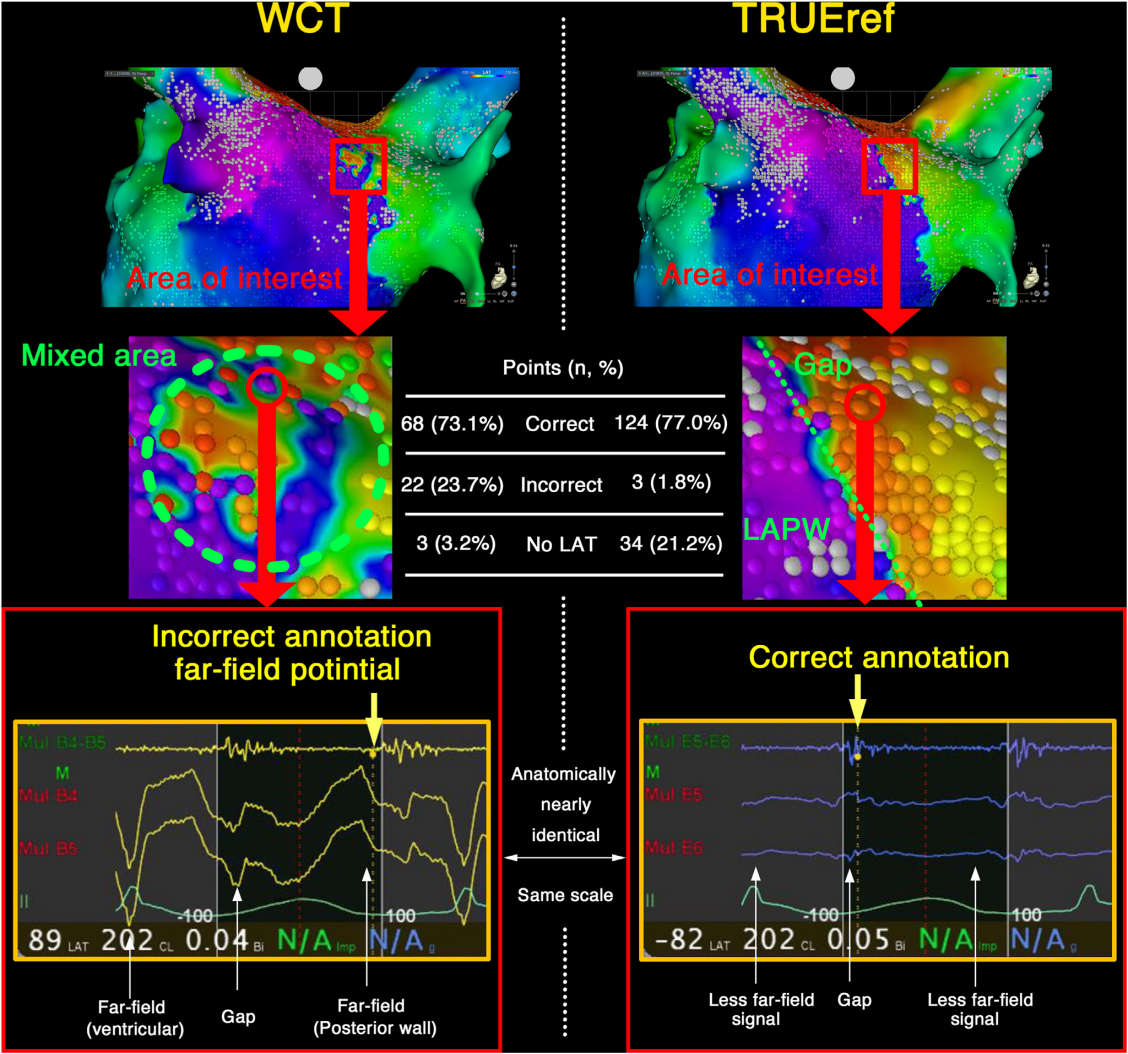
An enlarged view of the activation maps around the gap conduction is displayed. With the Wilson Central Terminal (WCT), the left atrial posterior wall (LAPW) and ventricular far-field potentials were recorded on the unipolar EGMs, and an incorrect annotation was observed. Furthermore, with the TRUEref, the near field potential was more accurately annotated because of the significant reduction in the far field potential, and the conduction gap was accurately depicted. A higher number of incorrect annotations were seen with MAP-W than with MAP-T, and the χ^2^ test demonstrated that the difference was statistically significant (MAP-W, 22 [23.7%] vs MAP-T, 3 [1.8%] points; *P* < .01).

**Figure 4:**
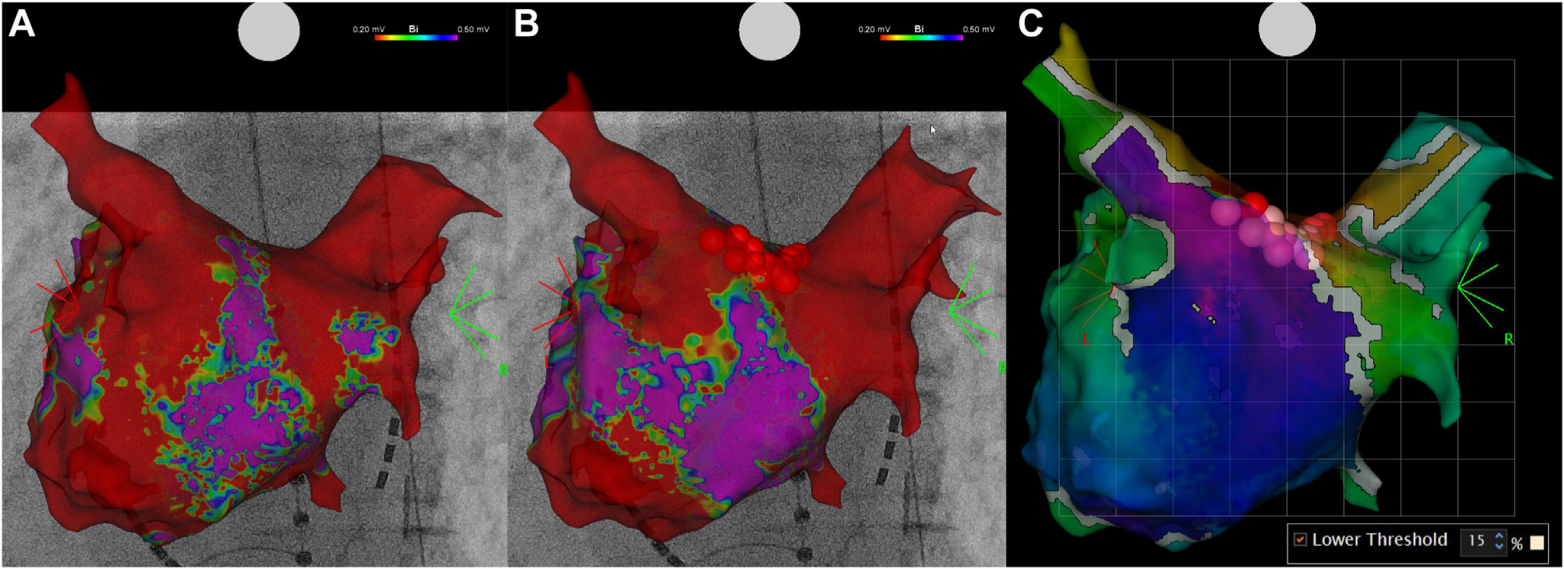
Before ablation, reconduction to the right pulmonary vein (RPV) was observed (**A**). A left atrial roof line was created, and additional ablation to the roof of the RPV successfully achieved re-isolation of the RPV (**B**). The successful ablation site corresponded to the conduction gap identified on the map using TRUEref, displayed by Early Meets Late with a lower threshold of 15% (**C**).

The LA activation maps were obtained twice by an experienced electrocardiologist (K.Y.) using an OCTARAY, once (MAP-W) with a conventional WCT and once (MAP-T) with the close indifferent electrode on the catheter shaft (Figure 2) to evaluate the usefulness of the TRUEref technology. The automatically determined local activation time (LAT) was nearly identical at most points, but differences were observed only at the conduction gap in the RPV. We conducted a detailed analysis of that area. MAP-W and the extended early meets late (EEML) technology with a lower threshold value of 15% showed the gap was depicted as a white line, indicating conduction block. However, with the TRUEref, the gap was visualized more accurately without any manual annotation. On detailed observation of the auto-annotation and LAT around the conduction gap, the LA posterior wall (LAPW) and ventricular far-field potentials were recorded in the EGMs, and an incorrect annotation was observed on the MAP-W. Especially in the area that appeared to have conduction gaps, there was a mixture of points annotated with LAPW far-field potentials and points annotated with continuous potentials at the conduction gap. As a result, the conduction gap was not adequately depicted (Figure 3). Conversely, the near-field potentials were more accurately annotated on MAP-T because of the significant reduction in the far-field potentials, and the conduction gap was accurately depicted. The numbers of points indicating a correct and incorrect LAT were counted, respectively. The accuracy of the annotations was assessed by 3 experienced electrophysiologists for consistency with the LAT of the surrounding points. There were a higher number of incorrect annotations with MAP-W than MAP-T, and the χ^2^ test demonstrated that the difference was statistically significant (MAP-W, 22 [23.7%] vs MAP-T, 3 [1.8%] points; *P* < .01). In MAP-T, numerous points were labeled as “No LAT” (MAP-W, 3 [3.2%] vs. MAP-T, 34 [21.2%]). Entrainment pacing from the LA anterior wall and posterior wall demonstrated the post-pacing interval was nearly equal to the tachycardia cycle length. A single linear ablation of the LA roof using an Qdot micro ablation catheter was conducted, and the AT was successfully terminated. The RPV was isolated after ablation of the gap depicted with MAP-T (Figure 4).

## Discussion

To the best of our knowledge, there have been no clinical reports comparing a WCT with a close indifferent electrode for activation maps of the same LA. Several interesting findings were observed in this case.

First, the close unipolar electrograms significantly reduced the large, sharp far-field potentials, and in areas with a low voltage and complex atrial electrograms, they accurately localized the lines of block and gaps along the ablation line in the clinical setting without any manual annotation. The accuracy of unipolar electrocardiograms is extremely important because the Wavefront annotation in the CARTO system depends on the maximal negative distal unipolar derivative of the electrocardiograms. The Close Unipolar Electrogram with the TRUEref technology enabled a more accurate annotation by minimizing the influence of the large far-field potentials in the current case. At sites where fragmented potentials with conduction gaps were recorded and annotated with MAP-T, large far-field potentials from LAPW conduction were observed with MAP-W at the same locations. In some points, these far-field potentials led to incorrect annotations (Figure 3). In addition, several technologies have been recently implemented in the CARTO 3 system to enable accurate mapping. For example, the accurate intracardiac pattern matching using the dual pattern matching method,^1^^,^^5^ combined with the far-field potential reduction by the TRUEref technology, may further enhance the diagnostic performance. Moreover, in TRUEref, not only did incorrect annotations decrease compared with WCT, but there were also a greater number of points identified as “No LAT.” Although the exact reason remains unclear, the filters and frequency settings involved in point acquisition were identical between MAP-W and MAP-T. Therefore, this phenomenon is likely attributable to the inherent differences between TRUEref and WCT. In low-voltage areas, a reduction in annotation of far-field potentials may lead to an increased number of points identified as No LAT.

## Limitations

In CARTO3 version 7.2, 2 MAPs cannot be created simultaneously using the TRUEref and WCT. Therefore, the anatomic locations of the acquired points do not completely coincide with MAP-T and MAP-W. A skilled electrophysiologist (K.Y.) created the maps in detail (MAP-T, 21,876 points; MAP-W, 14,230 points) to ensure that the points were in the same position as much as possible. The proximity of the indifferent electrode and the electrode on the catheter may affect the bipolar potential. The WCT is far enough away from all electrodes that the potential recorded with the WCT can be almost negligible. However, the close indifferent electrode of the Octaray is embedded at the root of the splines. The potentials recorded using the close indifferent electrode may affect the electrograms when the indifferent electrode is close to the atrial walls, but that was not fully investigated.

## Conclusion

The TRUEref technology reduced far-field potentials and contributed to a more accurate depiction of the conduction block.

## Disclosures

The ethical committee of Sendai Kousei Hospital waived the requirement for obtaining ethical approval because this research was neither a clinical study nor an animal experiment. Informed consent was obtained from the patient.
